# Cognitive functions explain discrete parameters of normal walking and dual-task walking, but not postural sway in quiet stance among physically active older people

**DOI:** 10.1186/s12877-024-05425-z

**Published:** 2024-10-19

**Authors:** Jimmy Falk, Daniel Eriksson Sörman, Viktor Strandkvist, Irene Vikman, Ulrik Röijezon

**Affiliations:** 1 Luleå University of Technology, Luleå, 97187 Sweden; 2 Umeå University, Umeå, 90187 Sweden

**Keywords:** Aging, Balance, Cognition, Dual-task, Executive functions, Gait, Intraindividual variability, Postural sway, Postural control, Processing speed

## Abstract

**Background:**

Postural control is dependent on the central nervous system’s accurate interpretation of sensory information to formulate and execute adequate motor actions. Research has shown that cognitive functions are associated with both postural control and fall risk, but specific associations are not established. The aim of this study was to explore how specific components of everyday postural control tasks are associated with both general and specific cognitive functions.

**Methods:**

Forty-six community-dwelling older adults reported their age, sex, physical activity level, falls and fall-related concerns. The following cognitive aspects were assessed: global cognition, executive functions, processing speed and intraindividual variability. Postural control was quantified by measuring postural sway in quiet stance, walking at a self-selected pace, and walking while performing a concurrent arithmetical task. Separate orthogonal projections of latent structures models were generated for each postural control outcome using descriptive and cognitive variables as explanatory variables.

**Results:**

Longer step length and faster gait speed were related to faster processing speed and less intraindividual variability in the choice reaction test. Moreover, longer step length was also related to less fall-related concerns and less severe fall-related injuries, while faster gait speed was also related to female sex and poorer global cognition. Lower dual-task cost for gait speed was explained by the executive function inhibition and faster processing speed. Postural sway in quiet stance was not explained by cognitive functions.

**Conclusions:**

Cognitive functions explained gait speed and step length during normal walking, as well as the decrease of gait speed while performing a concurrent cognitive task. The results suggest that different cognitive processes are important for different postural control aspects. Postural sway in quiet stance, step time and gait variability seem to depend more on physical and automatic processes rather than higher cognitive functions among physically active older people. The relationships between cognitive functions and postural control likely vary depending on the specific tasks and the characteristics of different populations.

**Supplementary Information:**

The online version contains supplementary material available at 10.1186/s12877-024-05425-z.

## Background

Accidental falls can have potentially devastating consequences on the health and wellbeing of a person as it might result in physical harm [1], development of fall-related concerns [2] and hence threaten both the identity and independence of a person [3]. Fall incidence increases with higher age and due to an aging population, the number of falls is expected to increase [4]. This trend can be mitigated by increasing our understanding of the postural control system to develop assessments to identify those with increased risk of falling and interventions to effectively reduce the risk [4]. An intriguing research branch has found that cognitive functions and postural control are associated, and it is suggested that cognitive assessments can improve fall-risk assessments [5–8]. However, the literature is heterogeneous, and more knowledge is needed regarding the specific relations between cognitive functions and postural control.

Postural control is the control of the body in relation to the tasks that are being performed and the current environmental demands and stems from interactions of sensory, cognitive, and motor functions [9]. A main objective of the postural control system is to control the center of mass in relation to one’s contact with the supporting surface [9]. This function is commonly assessed in standing by quantifying the sway of the body during different tasks, stances, and conditions. Usually, a force plate is used to quantify the movement of the center of pressure (COP), where decreased control over COP-movements indicates poorer postural control [11]. Aging comes with a natural deterioration of the human biology, which affects each of the underlying postural control functions [12–14]. This can for example be seen in older individuals who have larger postural sway than younger individuals [10]. Among older individuals, the cognitive systems seem important to control postural sway in quiet stance as sway increases in relation to increasing cognitive impairments [15]. This indicates that cognitive functions are important for postural control, even in rather simple postural tasks.

Walking is, for the majority, considered a simple task that requires little attention, although it is a highly complex skill comprising several discrete components that rely on many cortical and subcortical structures [16]. Among older patients, gait assessments are frequently conducted as part of clinical evaluations, where gait speed is a simple yet valuable test that has been shown to be indicative of both cognitive impairment [15] and increased risk of falling [17]. When separating gait into its different components, step length (heel-to-heel distance) and step time (time between successive heel strikes) are associated with different factors, indicating different control mechanisms [16]. Aside from quantifying the average of each component, it has proven valuable to quantify the variability of gait parameters, which also load into separate factors [18]. Increased step-to-step variability indicates lower levels of gait automatization, which has been found in populations with decreased mobility and impaired cognition [19]. By quantifying different gait components that rely on different control mechanisms, gait assessments become richer and can reveal features of core postural control mechanisms.

The quantification of specific gait components can explain a lot about walking behavior in a controlled setting. However, in everyday life, walking is often performed simultaneously with either cognitive or motor tasks. The simultaneous performance of different tasks often leads to impaired performance of either one or all tasks, i.e., dual-task cost. The bottleneck theory and the limited capacity theory are two of the most common theories to explain the dual-task cost phenomenon. The bottleneck theory proposes that neural networks that are involved in both tasks creates a bottleneck that limits performance. The limited capacity theory proposes that the attentional resources are limited and when two tasks are performed simultaneously, the resources are divided between the tasks. The more complex tasks and/or the more limited attentional resources, the higher the dual-task cost becomes [20]. Assessing dual-task walking is an established method for investigating motor-cognitive relations, where both physical and cognitive functions explain dual-task performance [21]. Compared to normal gait, assessing dual-task walking has been argued to be more sensitive for predicting falls among older adults [22].

To perform even simple postural tasks, the central nervous system performs numerous operations to gain perception of the body’s relation to the environment and generate appropriate motor commands [9]. Research has found several cognitive functions to be associated with postural control [23]. This is also observed in fall statistics, as older people with cognitive impairments have an increased risk of falling [6]. To identify people with deteriorating cognitive functions, i.e., mild cognitive impairment, instruments that consider several cognitive functions, i.e., global cognition, are used both in the clinic and in research [24]. When assessing for cognitive impairments, it is also valuable to ask about the person’s own subjective experiences regarding changes in their cognitive functions as it might detect pre-clinical cognitive decline [25]. Research has found that those with only subjective cognitive decline have similarly impaired dual-task performance as those diagnosed with mild cognitive impairment [26]. Although global cognition is associated with postural control, assessments of more specific cognitive functions seem to be better correlated with postural control function [23].

Among healthy older individuals with intact global cognition, the cognitive domain of executive functions has been shown to be associated with postural control performance [27] and the risk of falling [8]. Executive functions are described as higher-order functions responsible for controlled behavior, reasoning, and problem solving [28]. According to Miyake et al. [29], they can be divided into three separate but correlated, subcomponents: inhibition, updating, and shifting. Inhibition, sometimes also referred to as inhibitory control, involves the capacity to suppress dominant, automatic, or prepotent conflicting responses within a specific context [29]. Updating, also known as working memory updating, reflects the ability to quickly monitor and evaluate incoming information for its relevance to the task at hand and integrate this content with the information active in working memory when needed [29, 30]. Shifting, also sometimes referred to as cognitive flexibility or task switching, is often understood as the ability to quickly switch between different tasks or mental states [31]. The executive functions are related to the prefrontal cortex and its networks, which are especially sensitive to age-related changes resulting in early functional decline [13]. They are proposed to have specific functions to control attention and behavior during postural control tasks [7, 32]. However, the literature is heterogeneous, and recent meta-analyses of the topic have not been able to conduct analyses of how each sub-component (inhibition, shifting and updating) relate to specific postural control functions [23, 33]. An alternative approach that is more generic suggests that executive functions are, due to their susceptible nature, sensitive markers of general age-related changes, and the observed relations are rather correlative than causative [32]. More research is required to establish the complexities between the executive functions and postural control functions.

Cognitive functions, including executive functions, are, according to processing speed theory, dependent on the speed of cortical operations [34]. Processing speed is suggested to reflect the integrity of cortical structure, e.g., white matter density, and function, e.g., cortical activation patterns [35]. Processing speed is often assessed by measuring the time it takes to complete fairly simple tasks that do not require specific skills. Common processing speed assessments measure the time it takes to perform a motor response to a visual and/or auditory stimulus, i.e., reaction time, or the time it takes to perform simple matching and tracing tasks. The performance on different processing speed assessments is highly correlated, which indicates a general speed component that explains a large part of age-related cognitive decline [35]. A simple reaction time test has been shown to explain postural control strategies [36] and adaptability to repeated postural challenges [37] and has proven to be a valuable feature for fall risk assessments [38]. However, assessments that require more cognitive processing, e.g., choice reaction tests, seem to better represent cognitive processing speed [39]. A closely related measure of cognitive integrity and function is the quantification of response consistency of repeated simple operations, i.e., intraindividual variability (IIV). Processing speed and IIV seem to proxy slightly different aspects of cortical integrity and function, which is why it is suggested that both measures be considered [40].

The literature regarding motor-cognitive associations is heterogeneous, with inconsistent findings, and has not reached a consensus on how specific cognitive functions relate to specific postural control parameters. With sensitive measures that can divide each postural control task into discrete parameters and modeling that considers several cognitive functions for each postural control parameter, we can gain understanding of specific motor-cognitive relations for different postural control tasks.

## Methods

We adopted an explorative approach to address the aim of exploring if specific and generic cognitive functions are associated with specific components of postural control tasks frequently done in everyday life, i.e., postural sway in quiet stance, normal walking, and dual-task walking. We hypothesize that the three executive functions—inhibition, shifting and updating—that represent specific cognitive functions would show selective relationships to specific postural control parameters, especially during dual-tasking. Whereas measures of processing speed and IIV, that represent sensitive measures of general cognitive functions, would show more generic associations to more postural control functions.

### Participants

The objective was to recruit a sample of older adults with variable cognitive functioning but without conditions that clearly affect postural control or the ability to complete the test-protocol. As physical and cognitive variability is expected to increase with higher age, the age of 70 was set as the lower bound instead of the commonly used 65 years. Based on experience from previous research of similar samples, methods, and outcomes (e.g., [41, 42]), the required sample size was estimated to be 40–50 participants.

Recruitment was conducted via advertisement on region-based Facebook groups (with a total of approximately forty-thousand members) and via contact with local senior organizations that disseminated the information about the project (approximately 700 members). The ad entailed information about the project and invited persons aged 70 years or older with or without self-reported cognitive impairment to participate in the study. Additional inclusion criteria were the ability to stand and walk without assistance. The exclusion criteria were color vision deficiency; neurological diseases such as Parkinson’s disease or multiple sclerosis; a vestibular diagnosis; or diagnosed dementia.

### Descriptive data

The recruited participants were invited to the Human Health and Performance Lab – Movement Science at Luleå University of Technology. Height and weight were measured. Participant age, sex, years of formal education, and physical activity level were self-reported. The level of physical activity was divided into the following two queries: “During a regular week, how much time are you physically active in ways that are not exercise, for example walks, bicycling, or gardening? (0 minutes; 0–30 minutes; 30–60 minutes; 60–90 minutes; 90–150 minutes; 150–300 minutes or > 300 minutes)” and “During a regular week, how much time do you spend exercising on a level that makes you short-winded, for example running, skiing or fitness classes? (0 minutes; 0–30 minutes; 30–60 minutes; 60–90 minutes; 90–120 minutes or > 120 minutes)” [43].

Fall-related issues were probed with a 12-month self-reported fall history, noted on an ordinal scale: 0 = no fall; 1 = one fall; 2 = two or more falls. Twelve-month fall-related injuries were also reported via an ordinal scale: 0 = no injury; 1 = mild injury that did not require medical assistance; and 2 = severe injury that required medical assistance. Fall-related concerns were quantified by a composite of two instruments: the Falls-Efficacy Scale – International (FES-I), which is a 16-item questionnaire about concerns about falling during everyday activities [44], and the Fear of Falling scale, where the person rates their fear of falling on a 4-point Likert scale where 1 = not afraid and 4 = very afraid [45]. The combination of both instruments provides a more comprehensive assessment [45]. Hence, a person was considered to suffer from fall-related concerns if they either scored higher than 23 on the FES-I or higher than 2 on the Fear of Falling scale.

### Cognitive testing

Global cognitive functions were investigated with the Montreal Cognitive Assessment [46], which considers short-term memory, visuospatial abilities, executive functions, attention, concentration and working memory, language, and orientation to time and place. The results are summed to a score between 0 and 30, where 26–30 indicates normal cognition, 18–25 mild impairment, 10–17 moderate impairment and 0–9 severe impairment [46]. MoCA was developed in response to the unsatisfying validity of the standard screening instrument for global cognitive function, the Mini-Mental State Examination [24]. When comparing the two instruments MoCA is more sensitive in detecting mild cognitive impairment [47]. Subjective cognitive decline was assessed with a question suggested by Langa & Levine [48]: “Do you feel that your cognitive function has declined in the past years? For example, do you experience deterioration in any of the following functions:

- Memory, e.g., misplacing things?
- Speech, e.g., difficulty finding the right words when talking?
- Visuospatial tasks, e.g., parking a car?
- Concentration, e.g., when cooking and using different devices simultaneously?”

If the participant recognized themselves in any of the given examples, they were considered to experience subjective cognitive decline, as noted on a dichotomous scale.

The executive functions, inhibition, shifting and updating, were tested with the color-word Stroop test, Trail Making Test (TMT) and Adapted Digit Ordering Test (DOT-A), respectively. The Stroop task has been a staple in neuropsychological assessments for almost a century [49]. It is an easily administered test that provides a relatively pure measure of inhibition [50]. The Stroop task is divided into two parts. For part one (Stroop-1), the participants were shown a paper sheet with 40 stars colored red, blue, green, or yellow. They were asked to verbally name the color of each star as quickly as possible. If they said an incorrect color, they were asked to correct themselves before proceeding. The time to complete the task was measured with a stopwatch by the test leader. This color-naming part of the test mainly reflects processing speed [51, 52]. For the second part of the test (Stroop-2), a sheet with 40 color words with incongruent colors (e.g., the word green in red font) was presented. The task was to name the color of the font for each word as quickly as possible. Faulty responses were corrected before proceeding. This part of the test reflects working memory, inhibition, and processing speed [51]. Before each test, the participants performed a practice trial with five stars/words. The outcome “inhibition” was generated by calculating the time difference between the Stroop-2 and Stroop-1 tests [50].

The paper version of the TMT is one of the most used instruments to assess cognitive functions in postural control research [33] and specifically measure shifting ability [53]. It also consists of two parts: part A and part B. For part A, 25 numbers are scattered over a sheet, and the task is to draw a line between the numbers in ascending order as quickly as possible. TMT-A is mainly explained by processing speed [54]. For part B, half of the numbers are replaced by letters. The task is to draw a line that alternated between the letters and numbers in ascending order (e.g., 1-A-2-B…). If the participants made an error, they were asked to correct themselves. Before each trial, the participants performed a practice trial with eight numbers/letters. The time needed to complete TMT-B can be explained by working memory and shifting, although the time difference between completing part B and completing part A is regarded as a pure measure of “shifting” ability [53].

The DOT-A is designed to test the ability to withhold and manipulate information in working memory i.e., updating. The test leader read aloud a sequence of digits in random order at a pace of one digit per second. The participants then recited the numbers in ascending order. Each sequence length was tested with two different series. The test started with a sequence length of three digits, and the longest sequence was eight digits. The test ended when the participant incorrectly recited the numbers for both trials of a sequence length. The outcome was the total number of correct responses, with the maximum possible score being twelve [55]. Before the DOT-A test, the participant performed a test trial with a sequence of four digits.

Processing speed and IIV were assessed with simple and choice reaction time tests on a 14ʺ Dell Inc. Latitude 7400 laptop with an Intel(R) Core(TM) i7-8665U CPU @ 1.90 GHz (Dell Technologies Inc., TX, USA). The participants were seated by a table in front of the laptop so that the hands could rest on the keyboard with the elbows flexed at approximately 90°. The tests were programmed on PsychoPy® 2022.2.4 [56]. For the simple reaction time test, a gray screen with a white 18.7 cm solid circle was presented, whereupon a 4.5 × 4.5 cm black asterisk appeared on the screen at which the participant pressed the spacebar as fast as possible. For the choice reaction time test, the same background was presented, but either a 3.4 × 3.4 cm left, or right arrow appeared upon which the participant pressed a corresponding key. Before each test, a practice trial of 16 repetitions was performed. Each test trial consisted of 40 repetitions with semi-random intervals between two and four seconds. Key presses faster than 150 ms or slower than three standard deviations above the mean for that individual were excluded because they likely represented an erroneous attempt (pressing early or, e.g., missing a key) [57]. Both the mean reaction time and IIV were calculated for both tests. The IIV was calculated as the percentage of the coefficient of variation 100*(standard deviation/mean) [58].

### Postural control testing

Postural sway was assessed on a Kistler force plate (Kistler, Winterthur, Switzerland) with a sampling frequency of 100 Hz. The participants stood with the same self-selected stance width under four different conditions: on a firm surface with open and closed eyes and standing on a soft, six-centimeters thick, Airex mat (AIREX, Sins, Switzerland) with open and closed eyes. The gaze was directed at a spot at eye level on a wall four meters in front of the participant. Each trial was recorded for 30 s. The signal was filtered with a fourth-order low-pass Butterworth filter with a 10 Hz cutoff. The length of the sway path was calculated for each condition and normalized to participant height. Sway path is one of the most common sway measures that is valid to compare postural sway for different balance conditions within groups [10, 59] as well to capture difference and between groups [10, 11, 60].

Gait was assessed with a 4.27 m long GAITRite-electronic walkway (GAITRite; CIR Systems Inc., NJ, USA) [61]. The participant walked over the mat at habitual walking speed five times. Approximately 2.5 m of distance before and after the walkway allowed for sufficient acceleration and deceleration. Dual-task performance was assessed with the participant walking five more times over the walkway, while performing serial subtractions by threes, starting at 100. If the participant counted to zero, they would restart by counting from the number 101. Gait speed (m/s), average step time (s), and step length (cm), as well as the percentage of the coefficient of variation for step time and step length, were generated for both normal walking and dual-task walking. All variables were normalized to participant height. The dual-task cost for each walking variable was calculated accordingly: 100*(Normal Walking – Dual Task Walking)/Normal Walking [62].

### Analysis

The data were imported into Excel for preprocessing. For interpretability, selected variables were multiplied by -1 so that higher values on all cognitive and postural control tests reflected better performance. All variables were imported to SIMCA 17 (Sartorius AG, Göttingen, Germany). Separate orthogonal projection of latent structures (OPLS) regression models were generated for each postural control variable. The descriptive and cognitive variables were set as X-variables in each model. Each model was validated with permutation plots. A model was considered valid if both the explained variance (R2) and predictive ability (Q2) were positive and higher than those of any of the permutations [63]. Only the valid models are presented and analyzed.

## Results

**Fig. 1:**
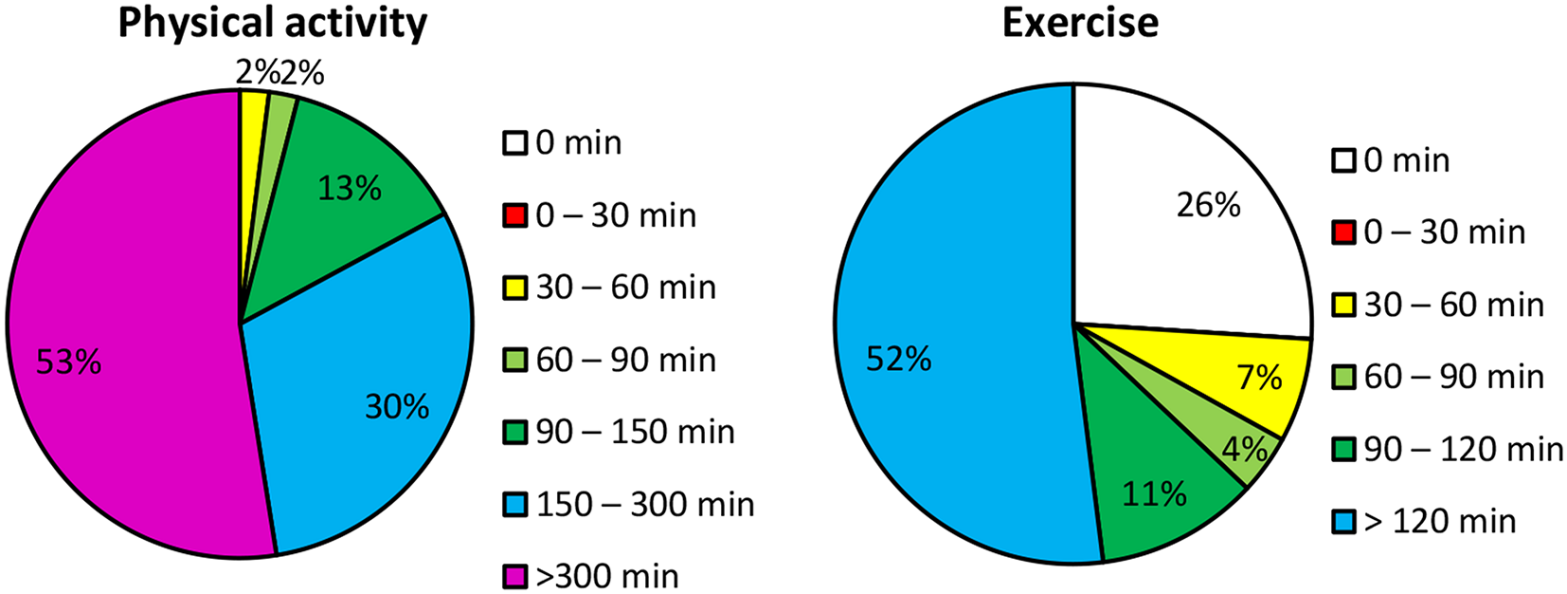
The percentage of participants that reported performing the corresponding level of physical activity and exercise each week

Forty-six individuals—30 women, with a median age of 78.5 years—participated in the study. Two participants were extreme outliers in the dual-task models and were excluded from those models. Five participants who failed to maintain an in-place strategy during the quiet stance test with their eyes closed on a soft surface were excluded from that variable. Descriptive data and the results from the cognitive assessments are presented in Table 1. The level of self-reported physical activity and exercise is presented in Fig. 1.

**Table 1:** Descriptive data of the participants and the results from the cognitive tests. Continuous data are presented in medians (Q1–Q3). Nominal data are presented in percentage of the total sample

Descriptive data		Cognitive tests	
Age (Years)	78.5 (72.8–79.0)	^a^MoCA	27 (25–28)
Sex (Women)	65%	MoCA < 26	33%
Weight (kg)	70.5 (62.1–82.0)	Subjective Cognitive Decline	58.7%
Height (cm)	165.8 (161.0–177.5)	^b^TMT-A (s)	29.8 (25.2–36.2)
Education (Years)	15 (13–17)	^c^TMT-B (s)	83.3 (63.3–113.3)
One fall	28%	^d^TMTB-A (s)	48.28 (37.3–78.0)
Two or more falls	9%	^b^Stroop1 (s)	27.08 (23.5–31.1)
Mild injury	22%	^e^Stroop2 (s)	49.25 (44.6–64.2)
Severe injury	7%	^f^Stroop2-1 (s)	22.15 (18.1–34.0)
FES-I	18.5 (17–24)	^g^DOT-A	5 (4–6)
FoF (1–4)		^b^Simple mean (ms)	318.7 (291.3–354.8)
1	50%	Simple IIV	17.0 (12.7–26.2)
2	24%	^b^Choice mean (ms)	547.3 (505.1–619.3)
3	22%	Choice IIV	15.3 (12.3–18.6)
4	4%		
Fall-related concerns	32.6%		

^a^Global cognition, ^b^processing speed, ^c^working memory and shifting ability, ^d^shifting ability, ^e^working memory, inhibition, and processing speed, ^f^inhibition, ^g^updating. Abbreviations: FES-I = Falls Self Efficacy – International; FoF = Fear of Falling scale; TMT = Trail Making Test; DOTA = Adaptive Digit Ordering Test; IIV = intraindividual variability

None of the quiet stance postural sway variables generated valid models and were not further analyzed. Non-normalized sway path for each postural sway condition is presented in Table 2. The models for normal walking gait speed and step length were valid, with explained variances of 0.627 and 0.445, respectively, and predictive abilities of 0.338 and 0.175, respectively. For dual-task performance, only dual-task gait speed generated a valid model with an explained variance of 0.617 and a predictive value of 0.255. Descriptive data for the calculated gait variables for normal walking and dual-task walking are presented in Table 3. Permutation plots from the validation process for each generated OPLS model are presented in Supplementary Material.

**Table 2:** Sway path in mm, not normalized to participant height, during 30 s of quiet standing. Five participants failed the condition of standing with closed eyes on an unstable surface, hence their data are not included for that condition

	Open eyes	Closed eyes
Stable Surface	393 (321–537)	619 (415–824)
Unstable Surface	1150 (974–1674)	2024 (1742–2662)

Data are presented in medians (Q1–Q3)

**Table 3:** Non-normalized data for normal and dual-task walking components. Two participants with extreme dual-task values are not included in this data

Gait variable	Normal walking	Dual-task walking
Gait Speed (cm/s)	132.29 (17.73)	114.39 (20.36)
Step Length (cm)	65.49 (7.46)	61.54 (7.88)
Step Time (s)	0.50 (0.04)	0.55 (0.07)
Step Length Variability (%CoV)	3.74 (1.37)	5.00 (1.62)
Step Time Variability (%CoV)	3.30 (0.92)	4.42 (1.29)

Data are presented with mean and standard deviation. %CoV = percentage of coefficient of variation (100*standard deviation/mean)

For normal walking, faster gait speed was associated with female sex, lower MoCA scores, and better performance on the Stroop-1 and Choice IIV tests. Individuals with less fall-related concerns and less severe fall-related injuries took longer steps. Longer steps were also associated with better performance on the Stroop-1 test and Choice IIV. The models and respective coefficients are presented in Table 4.

The dual-task cost for gait speed was lower for individuals who performed better on Stroop2-1 and who had faster simple and choice reaction times, see Table 4.

**Table 4:** Results from the OPLS-models, showing the coefficients for each variable for every valid model

	Normal Walking Gait Speed	Normal Walking Step Length	Dual-Task Cost Gait Speed
Model R2/Q2	0.627/0.338	0.445/0.175	0.617/0.255
**Descriptive Variables**			
Age	-0.14 (-0.4–0.12)	-0.16 (-0.34–0.02)	-0.12 (-0.29–0.05)
Sex	**-0.39 (-0.76 – -0.02)**	-0.14 (-0.29–0.01)	0.22 (-0.03–0.47)
Education	0.04 (-0.26–0.34)	0.01 (-0.17–0.19)	-0.13 (-0.47–0.21)
Physical activity	-0.04 (-0.31–0.23)	0.07 (-0.04–0.18)	-0.2 (-0.49–0.09)
Exercise	0.12 (-0.18–0.42)	0.06 (-0.08–0.2)	0.29 (-0.04–0.62)
Falls	0.08 (-0.21–0.37)	-0.07 (-0.19–0.05)	0.05 (-0.22–0.32)
Injury	-0.08 (-0.3–0.14)	**-0.15 (-0.21 – -0.09)**	0.1 (-0.16–0.36)
Fall-related concerns	-0.2 (-0.45–0.05)	**-0.18 (-0.35 – -0.01)**	0.28 (-0.02–0.58)
**Cognitive Tests**			
Subjective Cognitive Decline	0.01 (-0.26–0.28)	-0.03 (-0.18–0.12)	0.16 (-0.16–0.48)
^a^MoCA	**-0.2 (-0.38 – -0.02)**	-0.1 (-0.32–0.12)	-0.13 (-0.37–0.11)
^b^TMT-A	0.09 (-0.14–0.32)	0.13 (-0.03–0.29)	-0.01 (-0.26–0.24)
^c^TMT-B	-0.03 (-0.13–0.07)	0.03 (-0.11–0.17)	0.04 (-0.1–0.18)
^d^TMTB-A	-0.05 (-0.16–0.06)	0 (-0.14–0.14)	0.05 (-0.15–0.25)
^b^Stroop-1	**0.18 (0.05–0.31)**	**0.16 (0.05–0.27)**	-0.18 (-0.48–0.12)
^e^Stroop-2	0 (-0.21–0.21)	0.06 (-0.11–0.23)	0.08 (-0.1–0.26)
^f^Stroop2-1	-0.14 (-0.36–0.08)	-0.05 (-0.25–0.15)	**0.24 (0.11–0.37)**
^g^DOT-A	-0.04 (-0.27–0.19)	-0.02 (-0.18–0.14)	0.01 (-0.24–0.26)
^b^Simple Mean	-0.11 (-0.35–0.13)	-0.04 (-0.19–0.11)	**0.21 (0.02–0.4)**
Simple IIV	-0.02 (-0.15–0.11)	-0.05 (-0.22–0.12)	0.07 (-0.21–0.35)
^b^Choice Mean	-0.01 (-0.29–0.27)	0.02 (-0.12–0.16)	**0.22 (0.01–0.43)**
Choice IIV	**0.29 (0.04–0.54)**	**0.14 (0.05–0.23)**	0.19 (-0.14–0.52)

^a^Global cognition, ^b^processing speed, ^c^working memory and shifting ability, ^d^shifting ability, ^e^working memory, inhibition, and processing speed, ^f^inhibition, ^g^updating. Sex is coded with 0 for women and 1 for men. A fall, an injury, or fall-related concerns is coded with 1, and negative results coded with 0. Higher values on the cognitive tests indicate better performance. Significant coefficients are shown in bold font. Abbreviations: TMT = Trail Making Test; DOT-A = Adaptive Digit Ordering Test; IIV = Intraindividual variability

## Discussion

The aim of the present study was to explore the associations between generic and specific cognitive functions and specific postural control parameters in quiet stance, normal walking, and dual-task walking conditions. We hypothesized that the executive functions would show selective relationships to certain postural control measures, whereas processing speed and IIV would be sensitive but show more generic relations to several postural control functions. The findings are quite contrary to our hypotheses and will be discussed in the following sections.

The models explaining the components of gait speed and step length for normal walking were valid, whereas the models for step time and gait variability were not. Gait speed and step length were explained to a high degree by the same cognitive functions, i.e., Stroop 1, testing processing speed, and IIV, for the choice reaction test. These similarities are consistent with studies that have used factor analyses to group gait components, where gait speed and step length load on the same factor, “pace”, whereas e.g., step time and variability load on different factors [18, 64]. Both processing speed and IIV are non-specific measures thought to reflect cortical structure and function, e.g., the integrity of both gray and white matter, the function of neurotransmitter systems [65], and neural activation patterns [35, 66]. The Stroop-1 differs from the other processing speed tasks as it requires a verbal response while the other require motor responses. The results indicate that color naming is more complex as the average response time of naming the color of each star is 677 ms, compared to 318.7 ms and 547.3 ms for the simple and choice reaction test, respectively. This could be explained by the fact that, even for the same task, verbal responses are slower than motor responses, indicating that different neurological networks are engaged [67]. Additionally, rapid color naming is a less automized task that provides more response options to choose from, which makes the task less predictable [68].

The step length model showed that individuals with previous fall-related injuries and those suffering from fall-related concerns took shorter steps during normal walking, and the same tendency was observed for gait speed, although non-significant. This might show that those who suffer from fall-related concerns adopt a cautious gait with shorter step length [69]. Fall-related concerns might lead to activity curtailment [2] and have been shown to affect postural control [70] and fall-risk [71]. This highlights that fall-related concerns are important to consider when assessing postural control.

A finding that is both surprising and puzzling is that poorer global cognition [46] was associated with faster normal gait speed. This finding might reflect an unstable relationship between global cognitive function and casual walking in an active older population, which is also found in the literature [16]. This relationship should be further investigated while considering depression, which has a complex relation to cognitive impairments [48]. MoCA and MMSE are the most common instruments to measure global cognition in postural control research, which makes them valuable for comparing different samples. As screening instruments, they have benefits as they are fast to administer, but both lack detail and have a ceiling effect [47]. Thirty-three percent of the sample had a MoCA score < 26, indicating mild cognitive impairment, whereas 58.7% reported subjective cognitive decline. This discrepancy might also be explained by a ceiling effect and a lack of consideration of previous cognitive capacity. Hence, individuals with innate high cognitive capacity may experience substantial cognitive decline before MoCA can detect it. Furthermore, in a heterogeneous community-dwelling sample, the daily activities and cognitive challenges ought to be quite variable. Hence, mild cognitive impairment can become more or less apparent. Consequently, those that only face modest cognitive challenges might not experience any issues even though there is a substantial decline in cognitive function [72].

The dual-task cost for gait speed was explained by Stroop2-1 performance, which represents the executive function inhibition. This could indicate a specific relation, that the executive function inhibition is important for monitoring stimuli and controlling behavior during dual-tasking. However, other studies have not found inhibition, tested with the Stroop-task to be associated with walking while performing a visuospatial task [73], naming animals, or counting backwards by sevens [74]. With these conflicting results, we are reluctant to further speculate on specific mechanisms of this association. Dual-task performance was also explained by processing speed, quantified by both the simple and choice reaction test. This is in line with findings that processing speed measured with a choice reaction test correlates with both dual-task walking while either performing a secondary cognitive or motor task [75]. It is a reasonable finding that both executive functions and processing speed explain the same variables as executive function performance is mostly explained by processing speed [39].

Our models were not able to explain the step time and gait variability for normal walking. Both components seem to stand under little influence of cognitive functions. Step time is suggested to mainly be controlled on a subcortical level but might due to e.g., pathologies become more cortically regulated [76]. Our sample showed relatively low gait variability [18], indicating a high level of automatization, which requires minimal cognitive control [19]. Literature reviews show that, for healthy individuals, gait variability seems to be a rather stable measure throughout aging [77] and is poorly associated with cognitive functions [19]. Hence, the quantification of step time and gait variability might be best suited when assessing frailer populations, with either physical or cognitive pathologies.

Postural sway in quiet stance was not validly explained by any of the models. This finding is in line with the literature showing poorer relationships between cognitive functions and static postural control tests compared to dynamic postural control tests [23]. Postural sway is probably also controlled by different mechanisms for different populations. The sway of this sample was probably more influenced by the conditioning of e.g., sensory systems that were systematically disturbed by the four different conditions [78]. But in frailer populations cognitive functions might become more important as sway among those with intact cognition, mild cognitive impairment, and mild dementia is increased for each respective group [15].

We hypothesized that the executive functions would have specific relationships to certain postural control functions. Inhibition did show a relation to the dual-task cost for walking and counting backwards. However, considering conflicting previous research, and that inhibition only was associated with the most complex postural control task we can’t suggest that this is a specific relationship. We also hypothesized that measures of processing speed and IIV would be generic and explain several functions. However, rather specific relations emerged where the pace-factor in normal walking was explained by a verbal-response speed test (Stroop-1) and the IIV of motor responses to the choice reaction test, whereas the dual-task cost for walking speed was better explained by mean motor reaction time. This supports that both the mean reaction time and IIV should be considered as they capture different cognitive aspects [40].

Based on our findings, we propose that a choice reaction test providing both mean and IIV could be an easily administered instrument to proxy different aspects of cognitive functions that are associated with postural control functions [5]. However, future studies would have to validate this, provide normative data and evaluate if the inclusion of these measures improves the predictive ability of fall-risk assessments.

## Strengths, limitations and future research

Some strengths and limitations of this study should be addressed. We consider it a strength that we assessed several cognitive and postural control functions and their components. OPLS modeling is well suited for explorative research because it allows relatively many variables in relation to the number of observations and does not have problems with multicollinearity [79].

The test protocol has some limitations that we would like to address to facilitate future research. Each cognitive function would have been better represented by assessing the same function with several tests and statistically extracting the desired outcome that is shared among the tests [80]. Cognitive test results could be influenced by e.g., depression which is important to consider for a nuanced understanding [48, 81, 82]. More strenuous physical tests would facilitate the interpretations in relation to physical capacity. Additionally, postural control tasks that are both more challenging and more like everyday activities would probably display clearer relationships that are more relatable to fall risk.

Lastly, as described previously, the motor-cognitive relationships seem to differ among populations. this sample was exceptionally active, with 83% being physically active for more than 150 minutes a week and 52% exercising for two or more hours each week. This could challenge the generalizability of the results, as only 58% of the age-matched population reaches the same level of non-exercise physical activity [83]. A frailer sample would probably display a higher reliance on cognitive functions to perform fairly simple postural control tasks [81, 82].

## Conclusions

Gait speed and step length were explained by verbal processing speed and choice reaction IIV, whereas the dual-task cost for gait speed was explained by mean motor reaction time of a simple and choice reaction test and the executive function inhibition. Postural sway in quiet stance, step time, and gait variability seem to depend more on physical and automatic processes than on cognitive functions among physically active older adults. The relationships between cognitive functions and postural control likely vary depending on the specific tasks and the characteristics of different populations, which sets up a vast assignment for future research to understand.

## Electronic supplementary material

Below is the link to the electronic supplementary material.

Supplementary Material 1

## Data Availability

The datasets used and/or analyzed during the current study are available from the corresponding author upon reasonable request.

## Abbreviations

DOT-A: Adaptive Digit Ordering Test
FES-I: Falls Efficiency Score - International
FoF: Fear of Falling scale
IIV: Intraindividual variability, 100*(standard deviation/mean)
MoCA: Montreal Cognitive Assessment
OPLS: Orthogonal projections to latent structures
TMT: Trail Making Test
