# Supplementary material for: Cognitive functions explain discrete parameters of normal walking and dual-task walking, but not postural sway in quiet stance among physically active older people
Source: BMC Geriatr. 2024 Oct 19;24:849. doi: 10.1186/s12877-024-05425-z (PMC11490021; doi:10.1186/s12877-024-05425-z)
Supplement: Supplementary file 1 — Supplementary Material 1 [file 12877_2024_5425_MOESM1_ESM.docx]

**Supplementary Material**

Figure 1-14 displays permutation plots that was used to validate each model for each postural control variable. The permutation plots display the models explained variance (R2) and predictive ability (Q2) to the right. To the left it displays the R2 and Q2 values for one hundred generated models with the same X-matrix as the original model, but the Y-values has randomly been permuted. Hence, it displays if the model is better at predicting the Y-value than the permuted models. A model is thus considered valid if both the R2 and Q2 values are higher than the corresponding value of the permuted models (1).


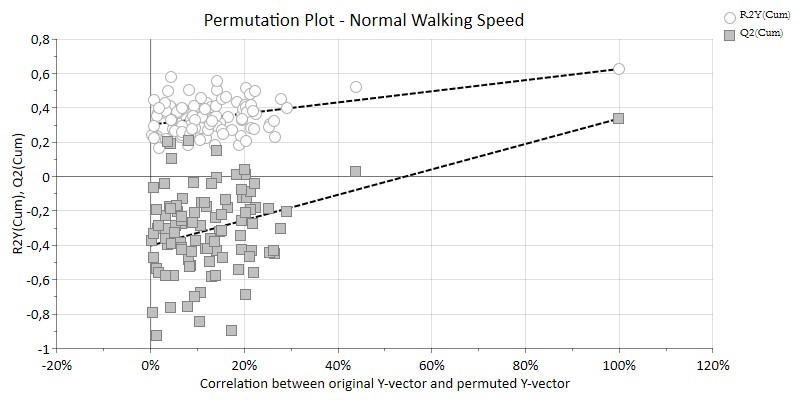


**Figure 1.** Permutation plot for the model “Normal walking gait speed”, showing a valid model.


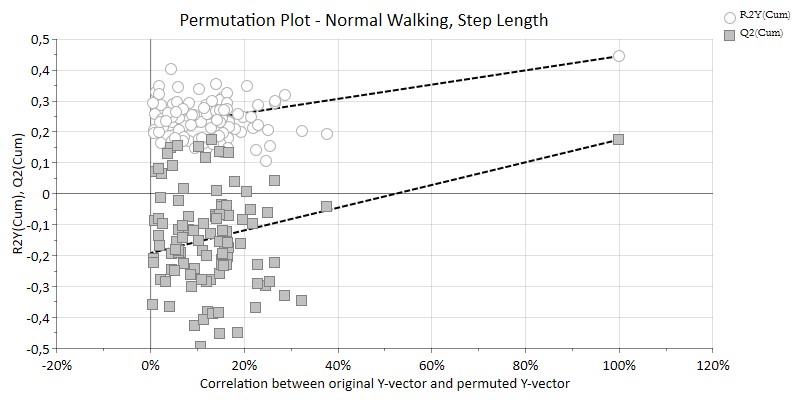


**Figure 2.** Permutation plot for the model “Normal walking step length”, showing a valid model.


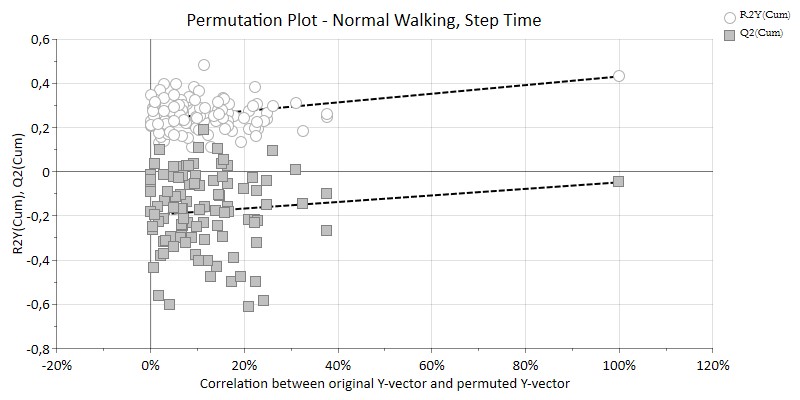


**Figure 3.** Permutation plot for the model “Normal walking step time”, showing a non-valid model.


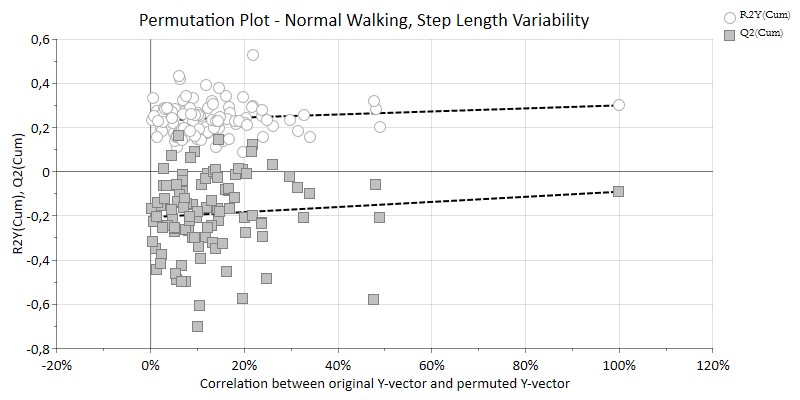


**Figure 4.** Permutation plot for the model “Normal walking step length variability”, showing a non-valid model.


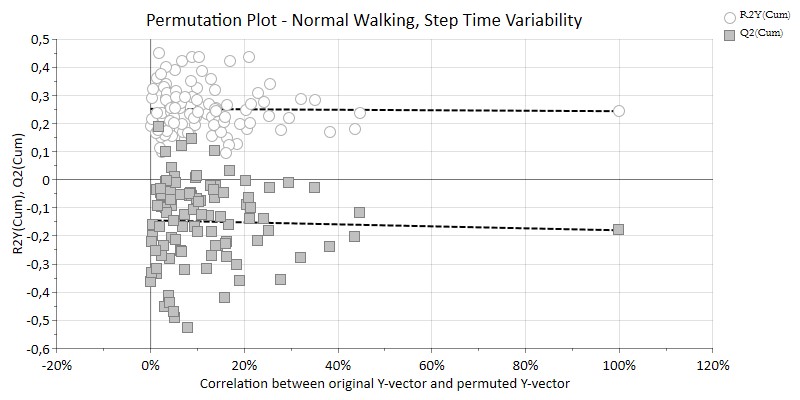


**Figure 5.** Permutation plot for the model “Normal walking step time variability”, showing a non-valid model.


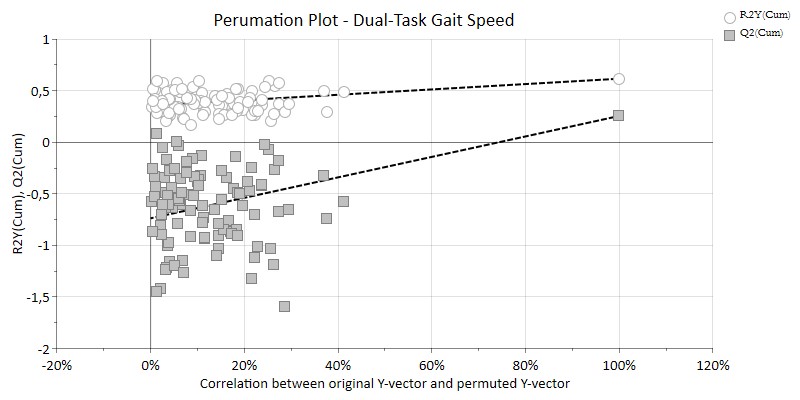


**Figure 6.** Permutation plot for the model “Dual-task gait speed”, showing a valid model.


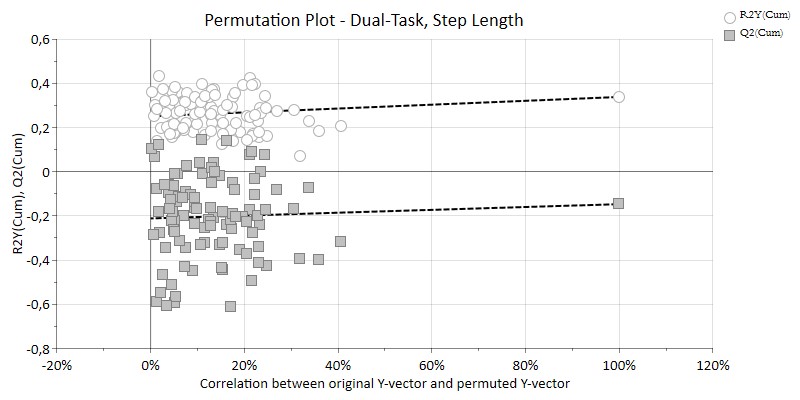
 **Figure 7.** Permutation plot for the model “Dual-task step length”, showing a non-valid model.


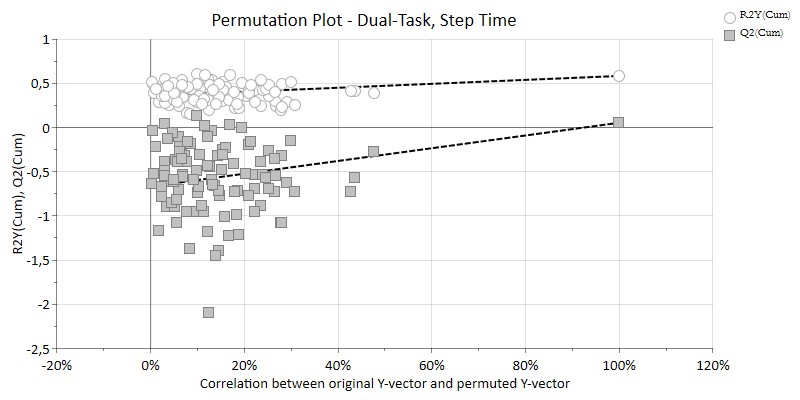


**Figure 8.** Permutation plot for the model “Dual-task step time”, showing a non-valid model.


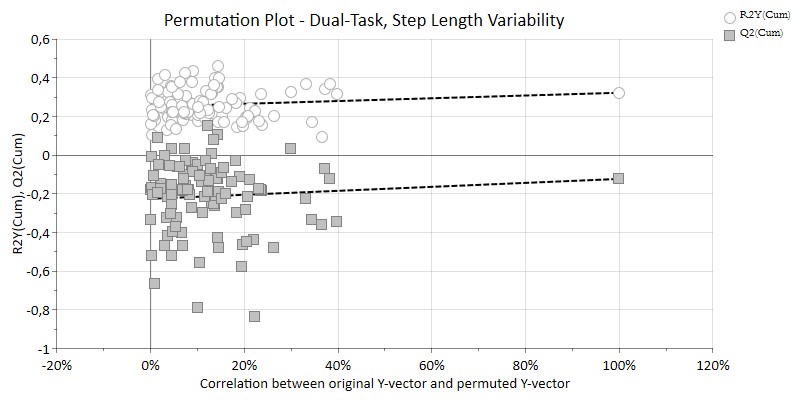


**Figure 9.** Permutation plot for the model “Dual-task step length variability”, showing a non-valid model.


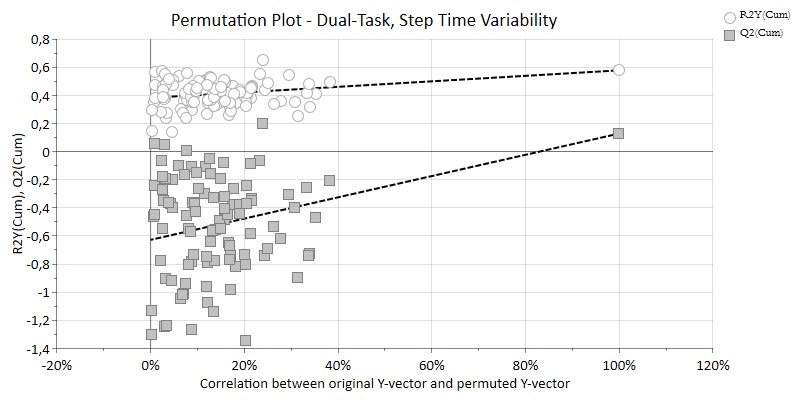


**Figure 10.** Permutation plot for the model “Dual-task step time variability”, showing a non-valid model.


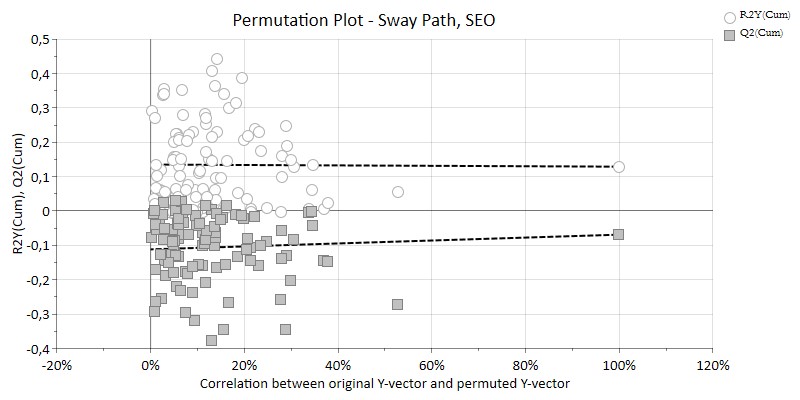


**Figure 11.** Permutation plot for the model explaining the sway path for the quiet stance condition with stable surface and open eyes, showing a non-valid model.


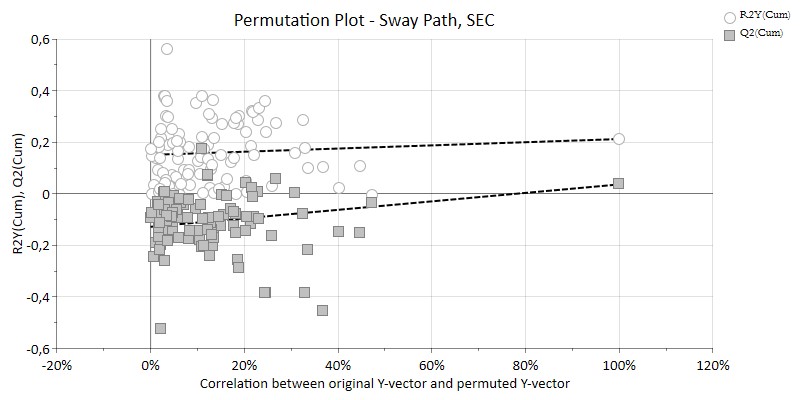


**Figure 12.** Permutation plot for the model explaining the sway path for the quiet stance condition with stable surface and closed eyes, showing a non-valid model.


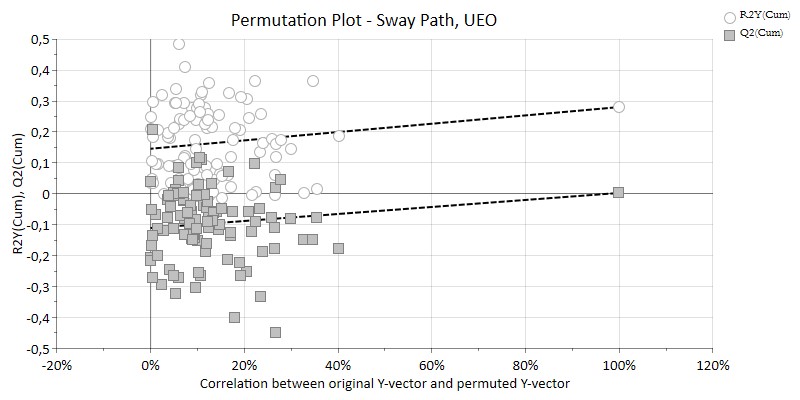


**Figure 13.** Permutation plot for the model explaining the sway path for the quiet stance condition with unstable surface and open eyes, showing a non-valid model.


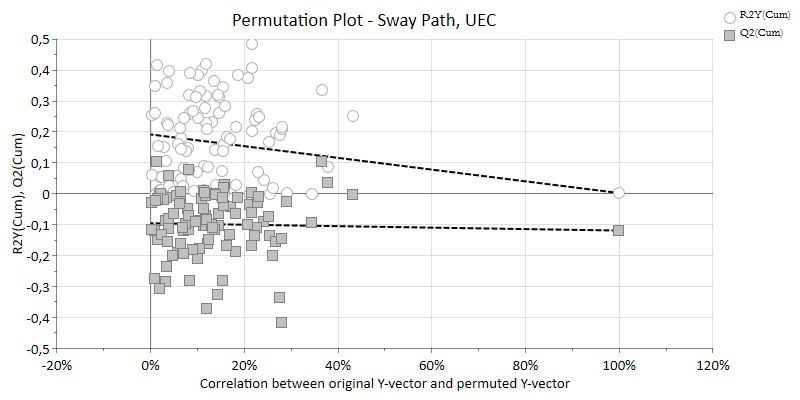
 **Figure 14.** Permutation plot for the model explaining the sway path for the quiet stance condition with unstable surface and closed eyes, showing a non-valid model.

**References**

1. Sartorius Stedim Data Analytics AB. *SIMCA 17 Advisor - Permutations Plot for PLS and PLS-DA Models*. Göttingen, Germany: Sartorius Stedim Data Analytics AB; 2021.
